# Neuroblastoma with superficial soft tissue mass as the first symptom: case reports with atypical ultrasonic image and literature review

**DOI:** 10.1590/1414-431X2023e12975

**Published:** 2023-12-11

**Authors:** Jiale Hu, Bei Xia, Xiuli Yuan, Haixing Chen, Fuxiang Ou, Longlong Huang, Lei Xu, Xia Feng

**Affiliations:** 1 Department of Ultrasound, Shenzhen Children's Hospital, Shenzhen, Guangdong, China; 2 China Medical University, Shenyang, Liaoning, China; 3 Department of Hematology and Oncology, Shenzhen Children's Hospital, Shenzhen, Guangdong, China

**Keywords:** Pediatric malignancy, Neuroblastoma, Atypical clinical manifestations, Soft tissue metastasis, Ultrasonography

## Abstract

Neuroblastoma is one of the most common tumors in children. Cases where an isolated soft-tissue metastasis mass is the initial symptom are rare, with only four such cases reported to date. We describe the imaging findings of ten cases of neuroblastoma patients in our hospital with superficial soft tissue mass (SSTM) as the primary symptom. The main ultrasound finding of SSTM was hypoechoic masses or scattered speck-like hyperechoic masses. However, when this type of SSTM is caused by soft tissue metastasis, the location is often atypical, and ultrasound findings are difficult to distinguish from other benign diseases. Therefore, this research should remind clinicians to recognize atypical presentations of this common childhood malignant tumor. Radiologists should also consider the possibility of neuroblastoma when finding this type of SSTM with atypical ultrasound features.

## Introduction

Neuroblastoma (NB) is the most prevalent extra-cranial solid tumor in childhood. It is most commonly found in the abdomen. The initial typical symptoms are fever, abdominal pain, and the presence of an abdominal mass (1). However, with diverse primary sites and a high risk of metastasis, the initial symptoms of NB are often atypical or similar to common benign mass diseases (2). It is uncommon for NB children to be hospitalized with superficial soft tissue mass (SSTM) as the primary symptom, and even rarer for children to present with SSTM due to NB metastasis (3,4). SSTM is more likely to occur in patients experiencing a recurrence or undergoing therapy. This uncommon clinical manifestation of NB is easily misdiagnosed, resulting in missed appropriate therapeutic opportunity. In this study, we identified ten cases of neuroblastoma confirmed by pathology in which SSTM was an early symptom. We retrospectively summarized clinical information and ultrasound features of ten clinical cases to provide a reference for future clinical diagnosis of NB.

## Material and Methods

### Enrolled patients

This retrospective study was approved by the Institutional Review Board of the Shenzhen Children's Hospital (No. 202211901), and the requirement for written informed consent was waived. We selected patients admitted to Shenzhen Children's Hospital with the initial symptom of local SSTM, which was finally diagnosed as NB based on pathological information. The start and end of data collection were September 2012 and December 2022, respectively. The enrollment criteria included pathology result demonstrating neuroblastoma, ganglionneuroblastoma, or ganglioneuroma and initial clinical symptom of external mass or lesion in musculoskeletal or subcutaneous soft tissue. The exclusion criteria were children with outer masses or lesions resulting from the subcutaneous spread of a primary abdominal lesion or experiencing a recurrence.

### Ultrasonic imaging and clinical information

Ultrasound (US) examinations were performed by experienced pediatric radiologists with systems from various manufacturers (GE LogiQ E9, USA; Mindray Resona 7, China) and 1 to 12 MHz linear-array transducers. Two experienced pediatric radiologists in consensus reviewed the US images of masses. Vascularity was assessed with color Doppler US.

Medical data included clinical information, laboratory test results, US imaging features, magnetic resonance imaging (MRI) information, and computed tomography (CT) image findings.

## Results

### SSTM due to the primary focus

In cases 1 to 3, the primary lesions were located in the neck (Supplementary Table S1). Patients presented with SSTMs but without fever, anemia, or other discomforts. The onset age of cases 1 and 2 was less than one year, while case 3 was over four years old. They all exhibited high neuron-specific enolase values. They were diagnosed with NB based on the pathological results.

In these three cases, we discovered hypoechoic masses (3/3) with sizes ranging from 4.9 to 7.4 cm in the ultrasonic images. These masses had an irregular shape (3/3), were sharply defined (2/3), heterogeneous (3/3), and densely speckled with hyperechoic areas (3/3). Case 2 exhibited abundant blood flow signals, while visible spots and strips of blood flow signals were observed in cases 1 and 3.

### SSTM due to metastases

Seven of the 10 patients diagnosed with NB had superficial soft tissue and lymph node involvement secondary to metastatic disease (Supplementary Table S1). There were a total of fifteen superficial masses. The size ranged from 2.5 to 7.0 cm.

**Figure 1:**
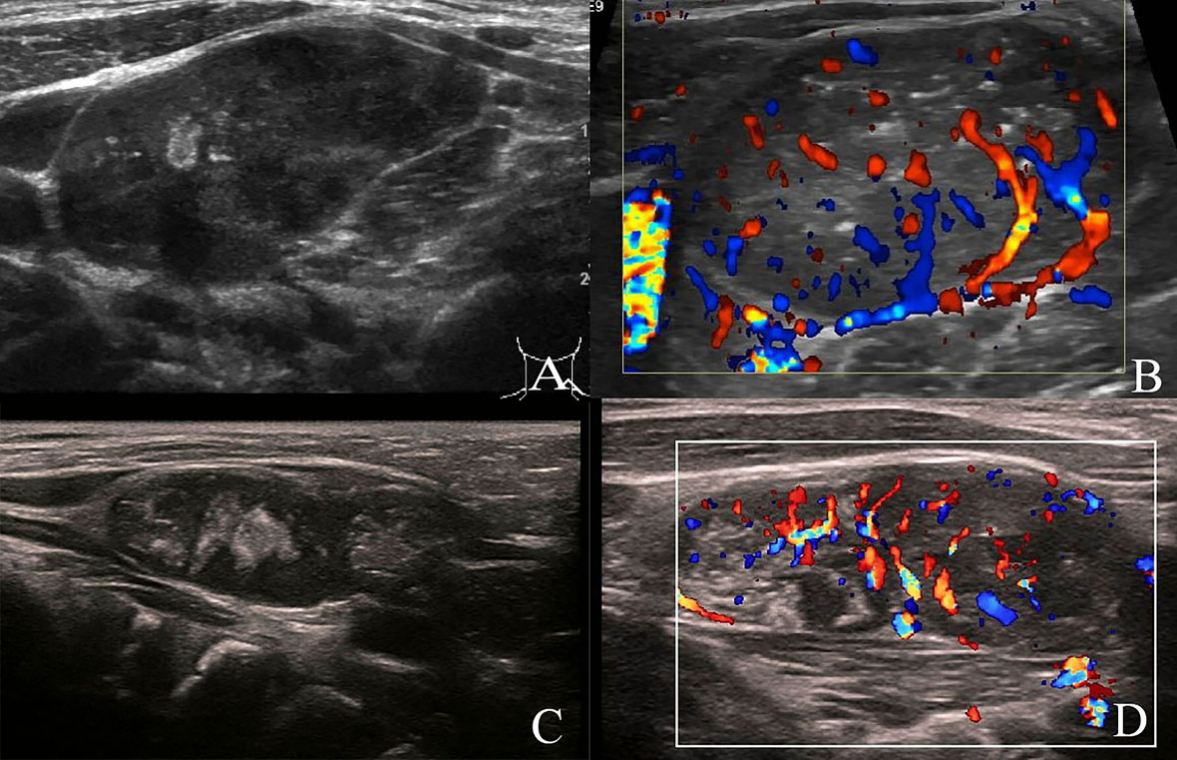
Ultrasound images of superficial soft-tissue masses due to lymphatic metastases: **A**, Female, 2y10m, left neck mass for four days. There were several enlarged lymph nodes in the left neck areas III and IV. The larger one was about 3.2×1.6 cm, oval in shape, with clear boundary, heterogeneous internal echo, patchy hyperechoic foci, and diffuse cortical thickening. **B**, Blood flow signals in enlarged lymph nodes are more abundant in the periphery than in the center. **C**, Female, 2y10m, left supraclavicular mass present for more than 4 months. Ultrasound image shows heterogeneous echogenic band with scattered bright echogenic spots and flaky dark area echoes. **D**, Color Doppler image shows hypoechoic mass with a dot-strip blood flow signal.

Cases 4 to 8 derived from lymphatic metastases, with four masses located on the left side of the neck, one mass in the left supraclavicular fossa, one mass in the right abdominal wall, and one mass in the suprasternal fossa. US indicated enlargement of lymph nodes (7/7) and heterogeneous (7/7), hyperechoic foci in the internal mass (5/7) (Figure 1A-D). Postoperative pathology identified case 4 and case 6 as ganglioneuroblastoma (GNB), while cases 5, 7, and 8 were diagnosed as NB.

**Figure 2:**
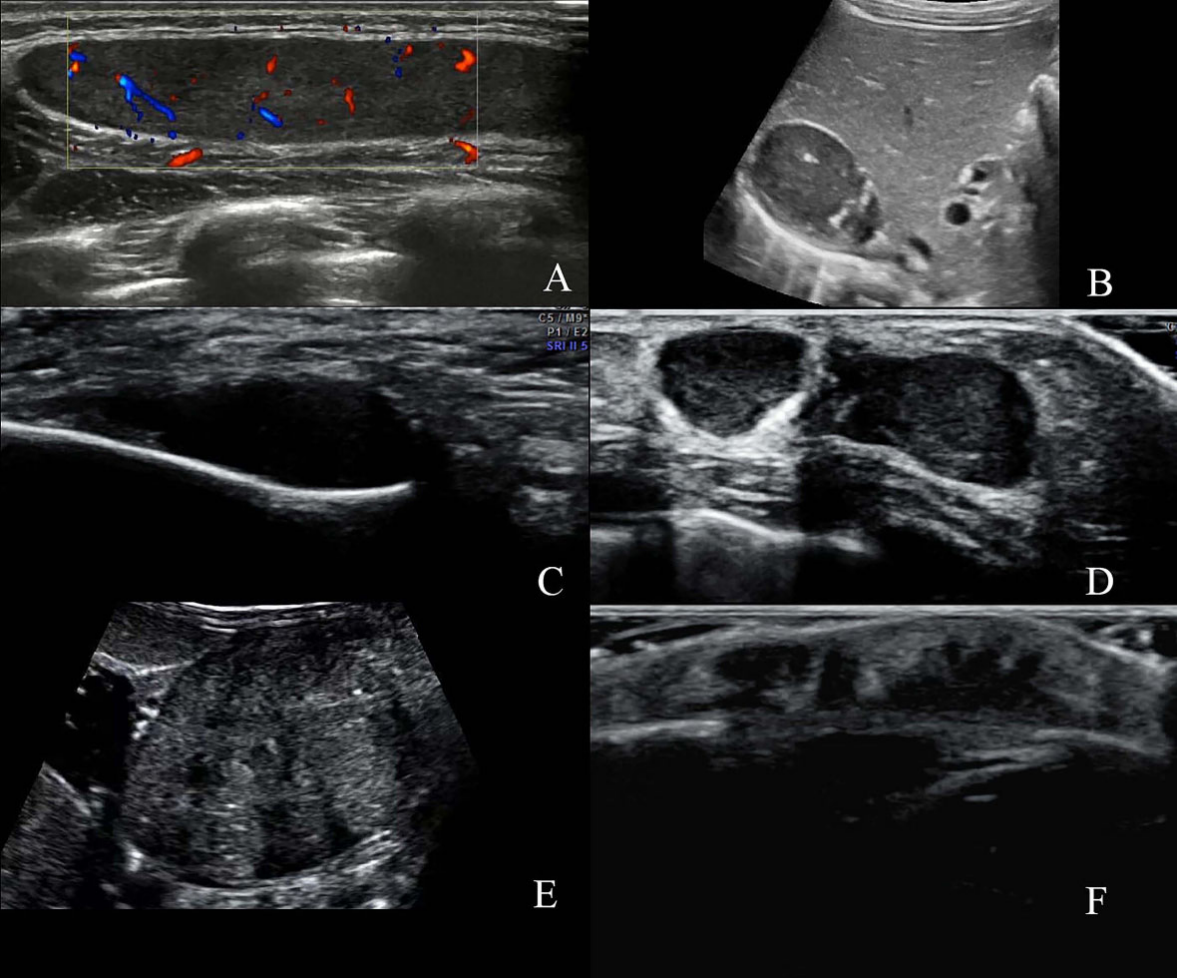
Superficial soft-tissue metastasis of a neuroblastoma. **A**, Female, 3y2m, right back mass for two years. A hypoechoic mass was observed between the subcutaneous fat layer and innermost intercostal area in the right back. This lump was well circumscribed with internal homogeneous echogenicity without patchy hyperechoic foci. Color Doppler flow imaging showed blood flow signals as dot strips. **B**, The adrenal area on the right side revealed a hypoechoic mass of 4.3×4.0×2.8 cm. The internal echo is hypoechoic, with hyperechoic foci. **C**, Female aged two months had numerous lumps in the whole body for one month. The front of the left ear showed a hypoechoic mass with a clear border and a homogeneous echogenic mass. **D**, The left chest wall indicated a hypoechoic lump with a clear border and heterogeneous internal echo. **E**, The left retroperitoneum presented a large substantial mass with a clear border and sparsely punctured calcification. The size was 8.0×5.5×4.5 cm. **F**, The top of the scalp showed an irregular hypoechoic mass with a clear border. It measured 1.0×1.0×0.6 cm. This lump had a heterogenic echo and did not indicate a prominent dark area or calcification. The blood flow signal in color Doppler flow imaging of the hypoechoic group was abundant.

Case 9 was a patient with soft tissue metastasis in the right back. The primary lesion was located in the right adrenal gland. She was initially misdiagnosed as having a benign fibroadenoma at presentation. Ultrasonography revealed a hypoechoic mass in the right back. Postoperative pathology identified the patient as having ganglioneuroblastoma (Figure 2A and B).

Case 10 had multiple metastases with multiple nodules in the fontanelle, in front of the left ear, and behind the right ear, on the right neck, on the chest wall, on the back, and inner side of the left thigh (primary focus was in the left retroperitoneum). The size of the nodules ranged from approximately 0.5 to 3.0 cm. The neuron-specific enolase level was 219.7 ng/mL. The patient did not exhibit symptoms such as fever, rash, abdominal pain, or other discomforts (Figure 2C-F). Postoperative pathology confirmed the diagnosis of NB.

## Discussion

Although clinicians currently conduct multidisciplinary treatment approaches for pediatric NB, the death rate is still 15% of the total mortality of malignant tumors in children. The long-term survival rate of high-risk children is only 40-50% (5). Early identification and diagnosis of neuroblastoma is of great importance in improving prognosis. Palpable and visible superficial soft tissue abnormalities are as an atypical initial presentation. However, there has been limited research on common sites of onset of SSTM. Our study reviewed ten cases with eighteen SSTMs. The SSTMs were located in the neck (38.9%), left supraclavicular fossa (11.1%), cephalo-facial (16.7%), back (11.1%), left chest wall (5.6%), subcutaneous soft tissue in suprasternal fossa (5.6%), left medial thigh (5.6%), and left abdominal wall (5.6%). Among the seven NB cases, the primary tumor site for six patients (cases 4, 5, 7, 8, 9, 10) was the retroperitoneum, while one patient (case 6) had tumors located in the posterior mediastinum. Compared to the three cases with primary lesions in the neck, these seven cases had a poorer prognosis.

Although some clinical diagnoses may require multiple imaging modalities, US is usually the primary tool for diagnosing children with SSTM as the initial clinical manifestation of NB (6,7). In this study, the three SSTMs that spread from primary lesions exhibited typical imaging features of NB, appearing as a hypoechoic lesion or a heterogeneous lesion with hyperechoic foci in the tumor (8). Differential diagnoses for these lesions include lymphoma, rhabdomyosarcoma, and other neurogenic tumors (9).

Five cases with the superficial soft tissue lesions from lymphatic metastasis had variable US appearances. These masses were usually enlarged, with loss of fatty hilum, a rounded rather than oval shape, hyperechogenicity, cystic change, calcifications, and peripheral vascularity. However, it was worth noting that case 6 was misdiagnosed as BCG (Bacille Calmette-Guérin) vaccine-induced regional suppurative lymphadenitis. The child received the BCG vaccination 4 months before his admission. He had no other clinical symptoms except for the SSTM in the left supraclavicular area. However, the enlarged nodules are usually located on the same side as the vaccination, occur mainly in male infants, and within 6 months of age. BCG vaccine-induced lymphadenitis appears as enlarged lymph nodes with liquefaction area and peripheral blood flow at color Doppler US in most cases (10). Lymphadenitis and malignant lymph nodes sometimes have similar ultrasound features, and their differentiation is challenging. Clinical features may provide more valuable clues than imaging findings, such as the speed of lesion progression, signs of infection, and effective anti-infective therapy, all of which are helpful in diagnosis.

Despite the fact that no research has analyzed the predictive value of US in NB cervical lymph node metastasis, Yang et al. (11) reported a meta-analysis that found that US was effective in diagnosing abnormal lymph nodes in the neck of patients with thyroid cancer. This meta-analysis indirectly proved that US performed better than CT in estimating cervical lymph node metastasis in a malignant tumor. Furthermore, most metastatic lymph nodes are in the lower third of the neck. The more reactive lymph nodes are in the upper part of the neck.

Furthermore, not all SSTMs show typical US presentations (12), especially masses from subcutaneous soft tissue metastasis. Soft tissue metastases are very rare, and the US appearance of cases 9 and 10 of this study was similar to other benign superficial soft tissue tumors. The grayscale ultrasonography presented a regular shape and well-defined structure and the internal echo of SSTMs was slightly heterogeneous and with no typical change, such as speckled calcification and variable internal vascularity at color Doppler US. Given a nonspecific US appearance, there are numerous diagnostic considerations, including myofibromas, hemangiomas, fibrous hamartomas, and neurofibromas (13). Case 9 underwent surgery because it was misdiagnosed as fibroadenoma. In ultrasonography, fibroadenomas usually appear as well-defined heterogeneous masses without internal vascularity. However, in this case, the echogenic intensity of the mass was lower than the expected echogenic level for a fibroadenoma, and no stripe-like hyperechoic region was observed.

In most situations, a combination of US findings and clinical history is adequate to guide appropriate management. However, metastatic tumors may manifest with a similar pattern of multiple soft tissue lesions. It is crucial to consider this possibility in children presenting with a palpable mass. Radiologists should not only emphasize the need for further imaging but also take advantage of US and extend the examination to the retroperitoneum, adrenal area, and posterior mediastinum to rule out the possibility of soft tissue metastasis. For cases such as case 9, with nonspecific sonographic and clinical findings, a biopsy is usually indicated.

Currently, only four NB cases have been reported where the initial presentation was SSTM due to soft tissue metastasis. Lumps were located on the thigh, malaris palpbra, trunk, and mammary gland. In addition, Avola et al. (14) reported a SSTM in the outer upper quadrant of the breast, in which the ultrasound showed an internal mottled calcification. The other 3 cases (15-17) showed only a hypoechoic mass on US. At first diagnosis, they were misdiagnosed as neurofibroma, lymphatic malformation, and hemangioma. In addition, one case with breast metastasis of neuroblastoma was reported by Feki et al. (18) in 2021, but this was not the initial manifestation in the child and showed only a hypoechoic mass on ultrasound.

### Conclusion

In conclusion, NB children with soft tissue metastases admitted with SSTM as the first symptom are extremely rare. When children present with ambiguous palpable lumps, a suspicion of metastatic neuroblastoma should be considered. The most appropriate strategy is to search for or identify the primary lesion by completing the retroperitoneal and mediastinal examination and combining it with clinical findings. If the SSTM involves deeper soft tissues, is large, or has a close association with a bony structure, or if a malignancy or high-flow vascular lesion is suspected, we should not avoid further cross-sectional imaging studies such as MRI and CT. In addition to oncologists, radiologists should also be aware of the various atypical clinical manifestations of neuroblastoma. This work is of great importance for early diagnosis and treatment of pediatric patients.

## Supplementary Material

Click to view [pdf].
